# *Lactobacillus rhamnosus* and *Staphylococcus epidermidis* in gut microbiota: in vitro antimicrobial resistance

**DOI:** 10.1186/s13568-022-01468-w

**Published:** 2022-10-03

**Authors:** Pamela Hindieh, Joseph Yaghi, André El Khoury, Ali Chokr, Ali Atoui, Nicolas Louka, Jean Claude Assaf

**Affiliations:** 1 Centre d’Analyses et de Recherche (CAR), Unité de Recherche TVA/Résistance aux Antibiotiques et Impact Industriel (RAII), Faculté des Sciences, Université Saint-Joseph de Beyrouth, Campus des sciences et technologies, Mar Roukos, Matn, Lebanon; 2 Laboratoire de Mycologie Et Sécurité Des Aliments (LMSA), Faculté Des Sciences, Université Saint-Joseph de Beyrouth, Campus des sciences et technologies, Mar Roukos, Matn, Lebanon; 3 Research Laboratory of Microbiology (RLM), Department of Life and Earth Sciences, Faculty of Sciences I, Lebanese University, Hadat Campus, Beirut, Lebanon; 4 Ecole Doctorale “Sciences Et Santé”, Université Saint-Joseph de Beyrouth, Campus des Sciences Médicales et Infirmières, Riad El Solh, Beirut, Lebanon; 5 Platform of Research and Analysis in Environmental Sciences (PRASE), Doctoral School of Sciences and Technologies, Lebanese University, Hadat Campus, Beirut, Lebanon

**Keywords:** *Lactobacillus rhamnosus* GG, *Staphylococcus epidermidis* 444, Biofilms, Lysozyme, GI tract, Antimicrobial resistance

## Abstract

The gastrointestinal tract is one of the most complex microbiological niches containing beneficial and non-pathogenic bacterial strains of which some may evolve into virulent under specific conditions. *Lactobacillus rhamnosus* GG is of the most known beneficial species with an ability to protect the intestine as opposed to *Staphylococcus epidermidis* 444 which causes serious health risks due to its high antimicrobial resistance. This study investigates first the survival and coexistence ability of *L. rhamnosus* GG, and *S. epidermidis* 444 at different pH levels. Subsequently, lysozyme's antimicrobial and antibiofilm effect on these two strains was elucidated before adding different concentrations of oxytetracycline hydrochloride antibiotic. Results showed that 50% inhibition of *L. rhamnosus* GG, *S. epidermidis* 444*,* and a co-culture of these planktonic strains were obtained respectively at a lysozyme concentration of 30, 18, and 26 mg/mL after the addition of ethylenediamine tetra-acetic acid (EDTA). At a pH of 7.5, mixing lysozyme (at IC_50_) and EDTA with oxytetracycline hydrochloride (700 μg/mL) showed an additional bactericidal effect as compared to its known bacteriostatic effect. Similarly, the addition of lysozyme to the antibiotic further increased the biofilm eradication of *S. epidermidis* 444 and *L. rhamnosus* GG where a maximal eradication of 70% was reached. Therefore, the potential development of new drugs based on adding a lysozyme-EDTA mixture to different types of antibiotics may be highly promising.

**Supplementary Information:**

The online version contains supplementary material available at 10.1186/s13568-022-01468-w.

## Key points

- Limiting antimicrobial resistance by adding lysozyme-EDTA mixture
- Converting bacteriostatic antibiotic to bactericidal by adding lysozyme-EDTA mixture
- Increasing biofilm eradication by adding lysozyme-EDTA mixture

**Supplementary Information:**

The online version contains supplementary material available at 10.1186/s13568-022-01468-w.

## Introduction

The human gastrointestinal (GI) tract is a complex system consisting of connected organs, forming a continuous passageway involved in providing the body with nutrients and energy sources by converting and absorbing food. The GI tract extends from the esophagus through the stomach, small intestine, and large intestine (colon) and ends in the anus (Gremel et al. 2015). Three successive regions of the small intestine are customarily distinguished proximally to distally: duodenum, jejunum, and ileum (von Rosenvinge et al. 2013). Since the GI tract is continually exposed to the outside, it has developed many protection systems such as the low pH in the stomach, the mucus layer that covers all the GI, a massive number of immune cells residing under the mucus layer, and finally an abundant mass of commensal microbes that colonize the GI tract (Hillman et al. 2017).

The human body is colonized by a complex ecosystem composed of commensal microbiota bacteria species. This mixture is obtained after birth and persists throughout life. Thus, due to their length and richness in nutrients, most of these microorganisms are heavily inhabited in the GI tract, where various diversity and density are observed (Ruan et al. 2020). Therefore, a gradient of bacterial colonization in the different interconnected organs of the GI tract according to the environmental variations in terms of physiology, digesta flow rates, substrate availability, host secretions, pH, and oxygen tension exists (Flint et al. 2012). Hence, the stomach is rarely colonized with less than 10^4^ bacteria per gram of stomach content. Going through the GI tract, the intestines harbor a significant number of bacteria, which increases to 10^7^–10^8^ CFU in the small intestine (Nishiyama et al. 2016).

However, bacteria throughout the GI tract can have several beneficial or adverse effects (Flint et al. 2012). Therefore, the state of the microbial community in terms of distribution, diversity, species composition, and metabolic outcomes affects the balance of benefit and harm for the host, so it can lead to better health or increase disease (Kim et al. 2007). Indeed, due to the high nutrient availability and constant influx of microorganisms, the GI tract forms an ideal site for the development of communities of microbial biofilms. Among the bacteria involved, Lactic acid bacteria (LAB), specifically *Lactobacillus* species, resides heavily as a beneficial microbe in the GI tract (Wang et al. 2014). As they are recognized as safe microorganisms, these bacteria are classified as probiotics (Tytgat et al. 2021). The *Lactobacillus rhamnosus* is currently detected as a dominant bacteria in the human gut (Nishiyama et al. 2016). Thus, due to its health benefits and ability to defeat intestinal pathogens, regulate intestinal flora balance, and maintain the intestinal barrier, more importance is given nowadays to this strain (Zhang et al. 2020; Matsubara et al. 2016). In addition to its antagonistic effect against harmful bacteria, this strain showed an ability to produce antimicrobial metabolites against many pathogens such as *Escherichia coli* (Li et al. 2020) *Salmonella enterica* (Zhang et al. 2018), and *Staphylococcus aureus* (Li et al. 2020). Furthermore, several studies also have shown that *L. rhamnosus* is efficient in reducing the bioavailability of mycotoxins in the GI tract (Assaf et al. 2019).

Another diverse bacteria group, *Staphylococcus* species, *Staphylococcus epidermidis*, are commensal bacteria frequently found in the human skin and mucous membranes such as the nose, throat, vaginal wall, and the gastrointestinal tract (Wang et al. 2014). While usually innocuous,

*S. epidermidis* can be an opportunistic pathogen (Kosecka-Strojek et al. 2020; Penelitian 2008). It is of great concern in nosocomial infections, especially in predisposed patients such as patients undergoing surgery or the immunocompromised (Pinheiro et al. 2015). Indwelling medical devices, like intravascular catheters, are considered a major vector for the infection with *S. epidermidis* biofilm (Otto 2014). Thus, leading to persistent and dangerous infections by these biofilms that are known to be highly resistant. Accordingly, they may resist the immune system and different antimicrobial agents including antibiotics that become useless (Ateba et al. 2010). Indeed, several genes coding for biofilm formation and antibiotic resistance are the key mechanism of *S. epidermidis* pathogenesis. These biofilms can disseminate into the bloodstream from their different formation sources. Within these sources, *S. epidermidis* has been found in the human GI tract (Brescó et al. 2017; Begot et al. 1996). Akinkunmi et al 2014, showed that *S. epidermidis* isolates from the intestinal tracts expressed high virulence factors, and an ability to biofilm-formation (Tamburini et al. 2018; Akinkunmi et al. 2014).

On the other hand, the overuse of antibiotics to kill pathogenic bacteria has endangered the effectiveness of these medications and attributed in many crisis, threatening global health by increasing medical expenses and mortality. Thereby, a widely recognized characteristic of antibiotic types is that they are either bacteriostatic such as tetracycline where they prevent bacteria from growing or bactericidal and used to kill the growing germs (Bernatová et al. 2013). According to several studies, oxytetracycline can be used to treat some pathogenic gut infections (Lovern et al. 2022; Ahn et al. 2021). However, most antibiotics offer short-term protection and are a source of antimicrobial resistance (Baquero 2021). For instance, a study conducted in 2019 showed that more than 100,000 deaths were related to antimicrobial resistance of many different strains (Murray et al. 2022). This global problem has led researchers to develop new strategies to combat these dangerous infections. A promising strategy to control the growth of pathogens may be by using enzymes.

Lysozyme is a safe secreted ubiquitous enzyme found abundantly in tears, saliva, human milk, mucus, and plasma that is considered as part of the innate defense system (Lu et al. 2010). Thus, it is defined as an antimicrobial enzyme that catalyzes the hydrolysis of 1,4-beta-linkages between N-acetylmuramic acid and N-acetyl-D-glucosamine residues in peptidoglycan, which is the major component of the gram-positive bacterial cell wall (Ercan and Demirci 2013). While lysozyme is known for its bactericidal properties, it has no cytotoxicity for human cells (Kim et al. 2020). It is important to mention that lysozyme has been approved by the FDA and in the European Union (European Food Safety Authority 2014).

In this study, the effect of adding lysozyme on planktonic or biofilms of *S. epidermidis* 444 and *L. rhamnosus* GG, singly or in co-culture was assessed. To imitate the GI tract pH level, the tests were conducted in a microplate-based model. The effect of adding different concentrations of oxytetracycline hydrochloride mixed with lysozyme and EDTA was also assessed. Therefore, the obtained results may reveal the behavior of the tested strains in the GI tract and their response to different antimicrobial agents. Thus, it may help in finding alternative solutions to limit antibiotic resistance by decreasing their used amounts.

## Materials and methods

### Bacterial strain and culture conditions

*L. rhamnosus* GG (ATCC 53103) and *S. epidermidis* 444 were purchased as lyophilized tablets from Microbiologics (St. Cloud, MN, USA). For planktonic antimicrobial assays, the strains were inoculated in Brain Heart Infusion broth (BHI) (Scharlab S.L., Spain) and cultured overnight at 37 ℃ under aerobic conditions (Parvekar et al. 2020). The bacteria were cultured in different media due to the presences of strains that requires a selective medium in which the growth is well established. Thus, *L. rhamnosus* GG was cultured in MRS broth (de Man-Rogosa-Sharpe) (Scharlab S.L., Spain), and *S. epidermidis* 444 was cultured in MHB (Mueller–Hinton Broth) (HiMedia Laboratories Pvt.Ltd., India). Both were cultured for 24 h under aerobic conditions at 37℃. The turbidimetric method was used to determine the bacterial cell concentration in MRS broth, MHB, and BHI (Begot et al. 1996). Thus, the absorbance was measured at 600 nm (OD_600_) using a spectrophotometer (Thermo Fisher Scientific, MA, USA), and the bacterial growth curves were constructed over a 24 h of incubation period (Additional file 1: Figs. S1, S2). The logarithmic value of bacterial concentration was also obtained by using the solid media counting method. An equation for calculation of the bacterial concentration was then generated with a compliant coefficient of determination (R^2^ = 0.999).

### Preparation of antimicrobial agents

The lysozyme (≥ 20,000 units/mg protein) was purchased from Vivantis (Vivantis Technologies, Malaysia) and dissolved in BHI medium to perform planktonic assays and in MRS and MHB for biofilm assays. Lysozyme stock solution (80 mg/mL) was prepared with and without adding 1 mM of Ethylenediaminetetraacetic acid (EDTA) (Sigma, St. Louis, MO, USA) to BHI at different pH: pH 2 (stomach); pH 6 (duodenum); pH 7.5 (small intestine) and pH 8.5 (colon) (Surat et al. 2018). For the biofilms, lysozyme with and without EDTA was assessed at pH 7.5. It is important to mention that all the used concentrations of lysozyme and EDTA (less than 2.5 mM) are safe and within FDA limits (European Food Safety Authority 2014; Wreesmann 2014). A stock solution of oxytetracycline hydrochloride purchased from sigma (St. Louis, MO, USA) was prepared at a concentration of 700 μg/mL in BHI for the inhibitory concentrations (IC) assay. A higher concentration of 2800 μg/mL was prepared in MRS and MHB for biofilm assays.

### Antimicrobial assays for planktonic bacteria

#### Half-maximal inhibitory concentration  (IC_50_) and minimum inhibitory concentration (MIC)

The half-maximal inhibitory concentration (IC_50_) represents the concentration of a drug or inhibitor (e.g. lysozyme, oxytetracycline hydrochloride) needed to inhibit the growth of a bacterial inoculum by 50% (Shen et al. 2021). Besides, the minimum inhibitory concentration (MIC) is defined as the lowest concentration of the substance at which there is no visible growth of a microorganism, as compared with control after an incubation time of 24 h (Kowalska-Krochmal and Dudek-Wicher 2021). These tests were performed using the micro-dilution method in a 96-well curved bottom non-treated microplate (Techno Plastic Products AG, Switzerland) as per guidelines of the National Committee for Clinical Laboratory Standards (Mogana et al. 2020). Then, series dilutions (1/2) of lysozyme (with and without 1 mM EDTA) in BHI were carried out in the wells, ranging from 80 mg/mL to 0.002 mg/mL with a final volume of 100 µL in each well (Sánchez et al. 2016). To create a highly resistant environment similar to the human gut, a high microbial concentration of 10^7^ CFU/mL was used (Mizunaga et al. 2005). Thus, a well-defined volume of the bacterial suspension (*L. rhamnosus* GG, *S. epidermidis* 444, or co-culture of both strains) was added to each well reaching a final volume of 100 μL with a bacterial concentration of 10^7^ CFU/mL. For the co-culture strains, the concentration of each of the inoculated bacteria inside the same well was equal to 5 × 10^6^ CFU/mL. Wells containing only the culture medium and the bacterial inoculum were used as a positive control. Wells containing only the culture medium and lysozyme were used as a negative control. Wells containing only the culture medium were used for sterility control. After inoculation, the plates were incubated at 37 °C for 24 h. Then, IC_50_ value was determined when the culture OD_600_ reaches half of the positive control OD_600_ (Umerska et al. 2018). When needed, further antimicrobial concentrations levels in between the performed dilutions were tested to find the exact inhibitory concentration value (IC_50_). The same protocol was repeated with oxytetracycline hydrochloride solution stock (700 µg/mL) that was serially diluted in BHI at a pH of 7.5 and within a range varying between 700 µg/mL and 0.17 µg/mL. A concentration of lysozyme (at IC_50_) mixed with 1 mM EDTA, was added to dilutions of oxytetracycline hydrochloride. Similar test was conducted with a mixture of oxytetracycline hydrochloride-lysozyme and EDTA (at IC_50_) in a 96-well curved bottom non-treated microplate.

#### Fractional inhibitory concentration (FIC)

The fractional inhibitory concentration (FIC) is a mathematical expression of the effect of antimicrobial agent combinations, calculated by dividing the MIC of each agent in the combination by the MIC of each drug alone. The FIC at the IC_50_ level (FIC_50_) were calculated for both drugs as follows:

FIC_50_ = IC_50_ drugs in combination **/** IC_50_ drug alone (Nunes et al. 2017)

$$\text{FIC}50 (\text{Lysozyme-EDTA}) = \frac{{\text{IC}}_{50 (\text{Lysozyme-EDTA}\;\text{with}\; \text{oxytetracycline}\; \text{hydrochloride}) }}{{\text{IC}}_{50 (\text{Lysozyme-EDTA})}}$$

$$\text{FIC}50 (\text{oxytetracycline}\; \text{hydrochloride}) = \frac{{\text{IC}}_{50 (\text{Lysozyme-EDTA}\; \text{with} \; \text{oxytetracycline} \; \text{hydrochloride}) }}{{\text{IC}}_{50 (\text{oxytetracycline}\; \text{hydrochloride})}}$$

ΣFIC is used to classify the nature of interaction: ΣFIC ≤ 0.5 indicates synergism; 0.5 < ΣFIC < 1 indicates additive effects; 1 < ΣFIC < 4 defines indifference and ΣFIC > 4 is considered as antagonism(Walsh et al. 1995). The ΣFIC_50_ index is the sum of FIC_50_ of the two agents tested:

$$\sum {\text{FIC}}_{50} = {\text{FIC}}_{50} \left( {\text{Lysozyme-EDTA}} \right) + {\text{ FIC}}_{50} \left( {\text{oxytetracycline} \; \text{ hydrochloride}} \right).$$

#### Minimum bactericidal concentrations (MBC)

The Minimum Bactericidal Concentration MBC is expressed as the lowest concentration of an antimicrobial agent which reduces the number of bacteria by 99.9% after an incubation time of 24 h (EUCAST 2000). MBC was determined after plating the contents of the wells and where no bacterial growth is visible on Petri dishes under sterile conditions. The plating was conducted at 37 °C under aerobic condition in MRS agar (Scharlab S.L., Spain) for *L. rhamnosus* GG and MHB agar (HiMedia Laboratories Pvt.Ltd., India) for *S. epidermidis* 444 at 48 h and 24 h respectively. For the co-culture, well-selected media are used to conduct the MBC; MRS agar was used to identify *L. rhamnosus* GG and the Baird Parker agar (Scharlab S.L., Spain) with egg yolk (Scharlab S.L., Spain) was used for the identification of *S. epidermidis* 444.

### Antimicrobial assays for bacterial biofilms

#### Preparation of bacterial suspension for biofilm assay

The bacterial pre-culture was prepared in MRS and MHB for *L. rhamnosus* GG and *S. epidermidis* 444 respectively. Then, it was incubated overnight at 37℃. The bacterial concentration of each strain was adjusted at 10^8^ CFU/mL in a tube of 15 mL (PLASTILAB, Lebanon). The bacterial suspension was centrifuged at 2500 rpm for 10 min. The supernatant was then discarded and pellets were washed with 1 mL of phosphate-buffered saline (PBS). The new suspension was centrifuged (2500 rpm, 10 min) and the supernatant was removed. The bacterial pellets were finally suspended in TSB (Scharlab S.L., Spain). This suspension was later used as an inoculum for the in vitro biofilm assay.

#### Preparation of the in vitro biofilm assay

For biofilm formation, 96-wells flat bottom treated microplates (Techno Plastic Products AG, Switzerland) were used. A volume of the adjusted bacterial suspension was inoculated in an appropriate medium to reach a final volume of 100 μL in each well with a bacterial concentration of 10^7^ CFU/mL. For the co-culture strains, the bacterial concentration of each strain was of 5 × 10^6^ CFU/mL. The biofilm was formed in MRS, MHB, and TSB at a pH of 7.5 respectively for *L. rhamnosus* GG, *S. epidermidis* 444*,* and their co-culture. Plates were incubated under aerobic conditions, without shaking for 72 h at 37 °C.

#### Minimal complete biofilm eradication concentration (MCBEC)

MCBEC_50_ was considered as the minimal drug concentration that can eradicated the microbial biofilm by 50% as compared to the positive control. The effect of lysozyme (80 mg/mL) with or without the addition of EDTA (1 mM) and oxytetracycline hydrochloride (2800 µg/mL) was tested on the formed biofilms. This test was only performed at a pH of 7.5 since *L. rhamnosus* GG and virulent *S. epidermidis* 444 biofilms are mainly formed in the small intestine (Tamburini et al. 2018; Reiter et al. 2013). After 3 days of incubation of *L. rhamnosus* GG, *S. epidermidis* 444*,* and co-culture of both strains, the supernatant was discarded and replaced by the prepared suspension using the same culture medium. The same protocols used for planktonic strains were also applied to the biofilm assays. Depending on the conducted test, serial dilutions (1/2) of lysozyme (80 mg/mL) and oxytetracycline hydrochloride (2800 μg/mL) were performed, with 100 μL as final volume in each well. Biofilms formed in new medium (without lysozyme, EDTA and antibiotic) were used as a positive control. Wells containing the tested additives were used as a negative control, and wells containing only medium were used for sterility test. After 24 h of incubation at 37℃, the supernatant was removed, and the biofilms were fixed by heating at 80 ℃ for 1 h. Then, the plates were stained with 100 µL of crystal violet solution (0.1%) added to each well and kept at room temperature for 10 min (Lim et al. 2020). The crystal violet was then discarded, and the biofilm was gently washed three times with distilled water to remove excess stain and non-adherent cells. The optical density was measured at 570 nm. Furthermore, a mixture of oxytetracycline hydrochloride-lysozyme and EDTA (at MCBEC_50_) was tested using 96-wells flat treated microplates. Same tests were performed as previously described above. The final volume in each well was of 100 μL with a fixed bacterial concentration of 10^7^ CFU/mL. After 24 h of incubation at 37 ℃, a staining was carried out using the same staining protocol and the optical density was measured at 570 nm.

### Statistical analysis

All tests were done in triplicate. To identify significantly different results, Two-way ANOVA was conducted using SPSS 19.0 (SPSS Inc., Chicago, IL, USA). The results with a *P* < 0.05 were considered statistically significant.

## Results

### Antimicrobial assays for planktonic bacteria

The inhibitory concentration IC_50_ was performed, and the optical density (OD_600_) was measured after 24 h of incubation. The obtained OD_600_ was converted into a percentage of viable cells. To note that different concentrations of EDTA varying from 1 mM to 2.5 mM were tested without observing any significant difference in the bacterial inhibition as compared to the control (data not shown).

#### Effect of increasing lysozyme concentration on *Lactobacillus rhamnosus* GG

**Fig. 1:**
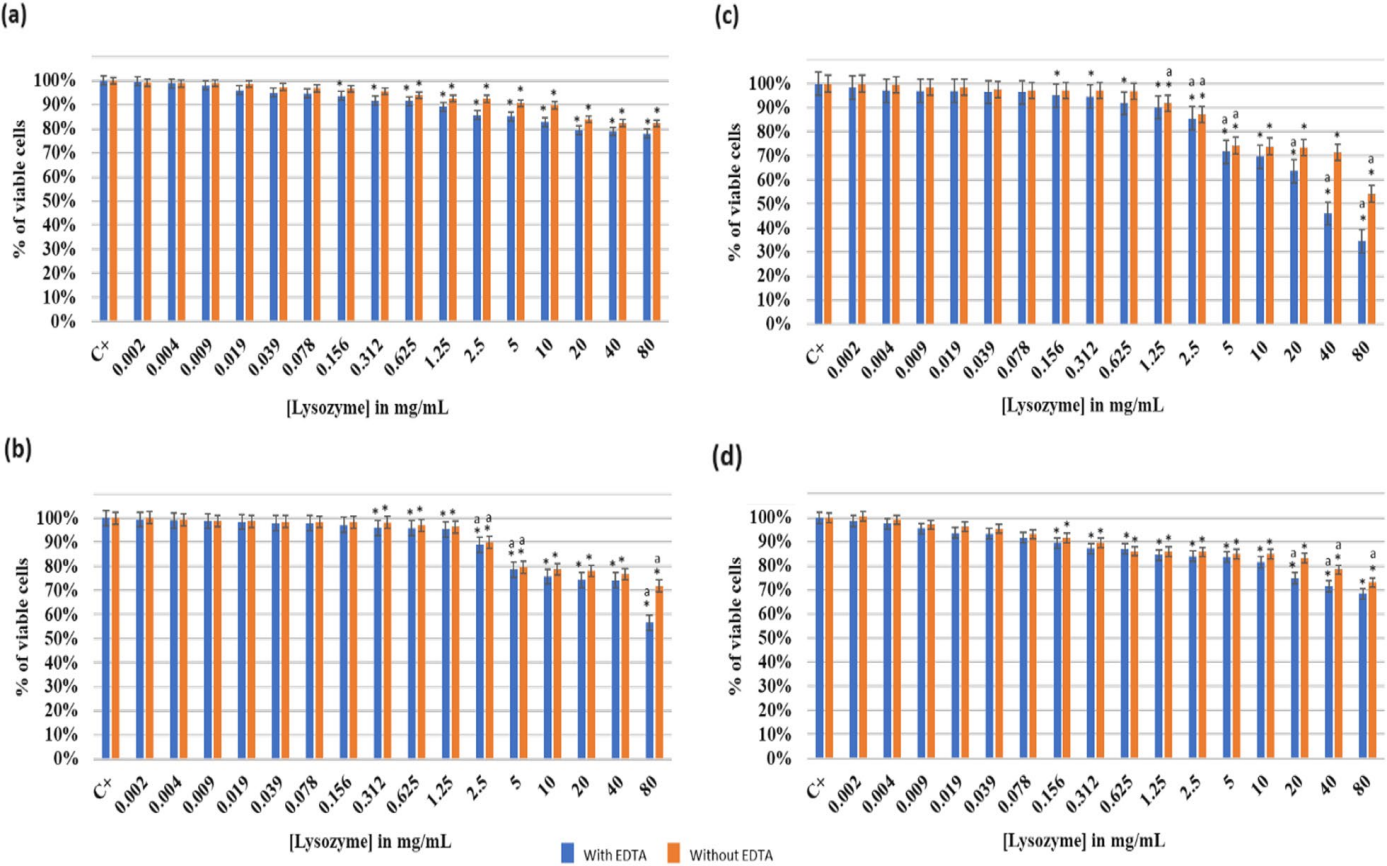
Effect of lysozyme with and without EDTA (1 mM) on Lactobacillus rhamnosus GG at different pH: **a** pH 2; **b** pH 6; **c** pH 7.5 and **d** pH 8.5. The results are mean values of three replicates. OD_600_ of the positive controls: C^+^(a) = 0.38; C^+^(b) = 1.36; C^+^(c) = 1.33;C^+^(d) = 1.32. (*) indicates a significant difference (P < 0.05) between each test and the positive control (without lysozyme). The lowercase letter **a** indicates a significant difference (P < 0.05) between each test and its preceding. Error bars represent the SD (standard deviation)

Figure 1 illustrates the impact of lysozyme with and without 1 mM EDTA on planktonic *L. rhamnosus* GG at different pH.

At pH 2, the addition of lysozyme addition did not have a wide effect on inhibiting viable cells (Fig. 1a). Thus, at a lysozyme concentration of 80 mg/mL, the percentage of viable cells decreased (P < 0.05) respectively from 100% (positive control) to 78% and 82% with and without adding EDTA. At this pH value, the growth of *L. rhamnosus* GG was limited compared to other pH values where the OD_600_ of the positive control increased from 0.38 at pH 2 to 1.33 at pH 7.5. At the same lysozyme concentration, a better inhibition effect was observed at pH 6 (Fig. 1b). Hence, an inhibition of 43% of the living cells with EDTA (57% viable cells) was reached. On the other hand, a higher percentage of cells (72%) remained viable without EDTA. However, lysozyme with EDTA exhibited its maximum antimicrobial activity at pH 7.5 (Fig. 1c). This gradual decrease occurred at a 1.25 mg/mL lysozyme concentration and beyond. At this concentration, a considerable drop (P < 0.05) of viable cells was observed to finally reach 35% at a concentration of 80 mg/mL. In contrast to other tested pH, a MIC was observed at 80 mg/mL of lysozyme (with EDTA) where an IC_50_ was detected at 30 mg/mL of lysozyme with EDTA. At a pH of 8.5 (Fig. 1d), a progressive decrease of cells viability was observed till reaching a 32% of bacterial growth inhibition (P < 0.05) at a lysozyme concentration of 80 mg/mL with EDTA. It is quite noticeable that increasing lysozyme concentration had a less significant effect at pH 8.5 compared to 7.5.

#### Effect of increasing lysozyme concentration on* Staphylococcus epidermidis* 444

**Fig. 2:**
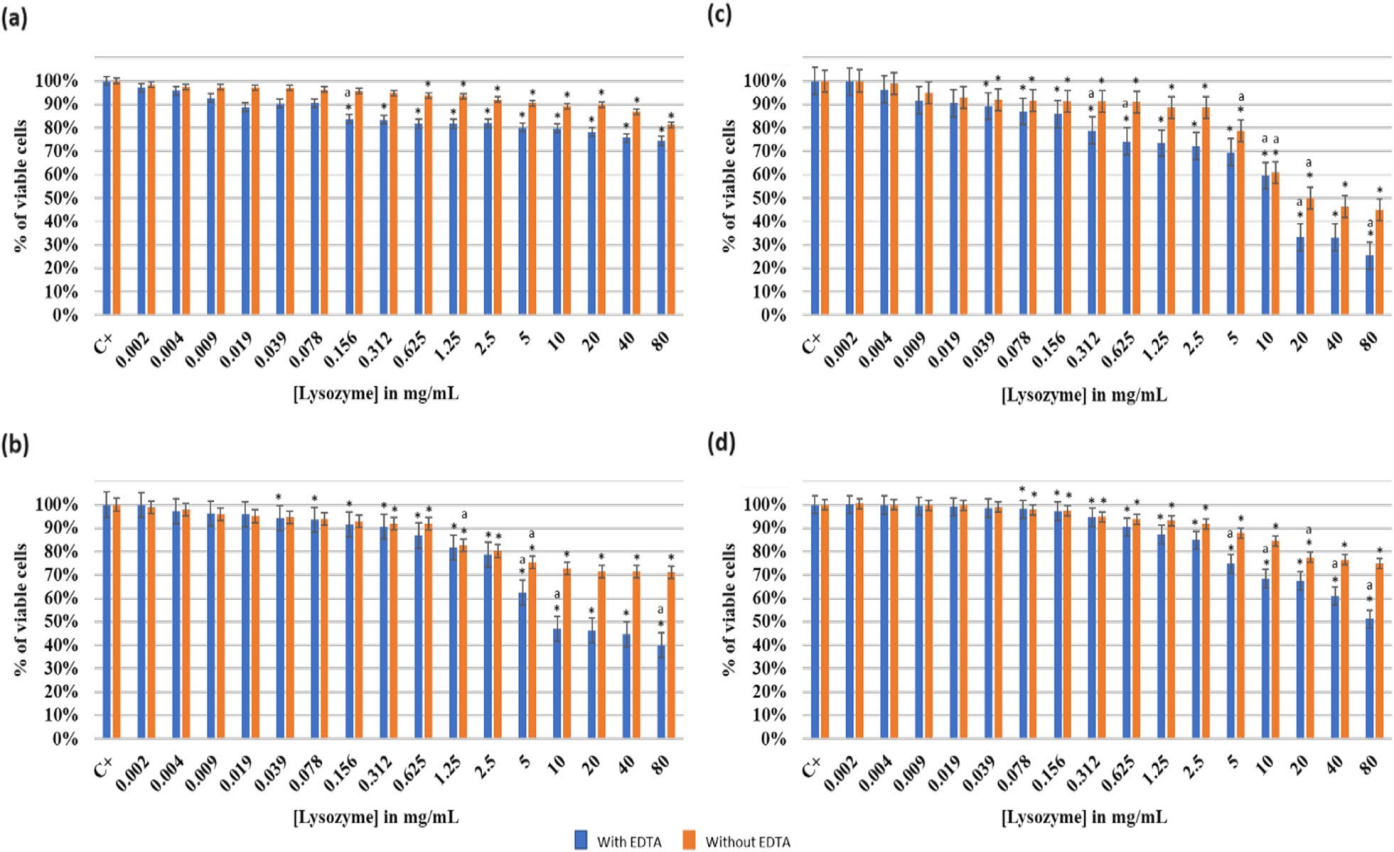
Effect of lysozyme with and without EDTA (1 mM) on *Staphylococcus epidermidis 444* at different pH: **a** pH 2; **b** pH 6; **c** pH 7.5 and **d** pH 8.5. The results are mean values of three replicates. OD_600_ of the positive controls: C^+^(a) = 0.45; C^+^(b) = 1.03; C^+^(c) = 1.30;C^+^(d) = 1.34. (*) indicates a significant difference (P < 0.05) between each test and the positive control (without lysozyme). The lowercase letter **a** indicates a significant difference (P < 0.05) between each test and its preceding. Error bars represent the SD (standard deviation)

The antimicrobial activity of the lysozyme with and without 1 mM EDTA on planktonic *S. epidermidis* 444 at different pH is highlighted in Fig. 2.

At pH 2, *S. epidermidis* 444 underwent a decrease (P < 0.05) in viable cells respectively from 100 to 74% (with EDTA) and to 81% (without EDTA) at a lysozyme concentration of 80 mg/mL (Fig. 2a). A better effect of lysozyme was observed at pH 6 (Fig. 2b). Accordingly, significant inhibition of viable cells growth (P < 0.05) was progressively expressed to finally reach 40% (with EDTA) and 71% (without EDTA) respectively at the same lysozyme concentration (80 mg/mL). Thus, a MIC was observed at a concentration of 40 mg/mL of lysozyme (with EDTA), and the IC_50_ was detected at 22 mg/mL of lysozyme (with EDTA). Although, lysozyme’s optimal inhibition effect was reported at a pH of 7.5 (Fig. 2c). Therefore, at a concentration of 10 mg/mL of lysozyme (with EDTA) a highly significant drop (P < 0.05) occurred. Thus, the percentage of viable cells decreased from 100 to 60% and kept decreasing till reaching 25% (with EDTA) and 46% (without EDTA) at a lysozyme concentration of 80 mg/mL. However, the MIC was visually observed at a lysozyme concentration of 40 mg/mL (with EDTA). The IC_50_ was determined at 18 mg/mL (with EDTA) and 26 mg/mL (without EDTA) of lysozyme (Table 1). Moreover, a progressive drop in the density of viable cells was observed at a pH of 8.5 (Fig. 2d), where a significant decrease (P < 0.05) by 51% was detected at 80 mg/mL of lysozyme with EDTA. A MIC was observed at 80 mg/mL of lysozyme with EDTA, and the IC_50_ at 50 mg/mL. However, with the same lysozyme concentration and without EDTA, 75% of the cells remained viable.

**Table 1:** Comparative table between the different MIC, IC_50_ and MBC of *Lactobacillus rhamnosus* GG, *Staphylococcus epidermidis* 444 and the co-culture of both strains

	Inhibitory Concentration (IC50)									
	Lysozyme (mg/mL)								Oxytetracycline hydrochloride (μg/mL)	
	With EDTA				Without EDTA				Without lysozyme-EDTA	With lysozyme-EDTA
	pH2	pH6	pH7.5	pH8.5	pH2	pH6	pH7.5	pH8.5	pH 7.5	
*L. rhamnosus* GG	ND	ND	30	ND	ND	ND	ND	ND	152	90
*S. epidermidis* 444	ND	22	18	50	ND	ND	26	ND	130	1.22
Co-culture strains	ND	ND	26	ND	ND	ND	ND	ND	ND	10.93
*L. rhamnosus* GG	ND	ND	80	ND	ND	ND	ND	ND	700	ND
*S. epidermidis* 444	ND	40	40	80	ND	ND	ND	ND	700	ND
Co-culture strains	ND	ND	80	ND	ND	ND	ND	ND	700	ND

	Minimum Bactericidal Concentration (MBC) at pH 7.5	
	lysozyme-EDTA (IC_50_) (mg/mL)	Oxytetracycline hydrochloride (μg/mL)
*L. rhamnosus* GG	30	700
*S. epidermidis* 444	18	700
Co-culture strains	26	700

	Minimal Complete Biofilm Eradication Concentration (MCBEC50) at pH 7.5			
	Lysozyme (mg/mL)		Oxytetracycline hydrochloride (μg/mL)	
	With EDTA	Without EDTA	Without lysozyme-EDTA	With lysozyme-EDTA
*L. rhamnosus* GG	50	66	ND	1144
*S. epidermidis* 444	26	30	ND	24
Co-culture strains	30	ND	ND	1464

*ND* indicates that MIC, IC_50_ and MCBEC_50_ was not detectable.

Different MCBEC_50_ of *Lactobacillus rhamnosus* GG, *Staphylococcus epidermidis *444 biofilms and the co-culture of both strains were also compared. All tests were performed at pH 7.5

#### Effect of increasing lysozyme on the co-culture of planktonic *Lactobacillus rhamnosus* GG and *Staphylococcus epidermidis* 444

**Fig. 3:**
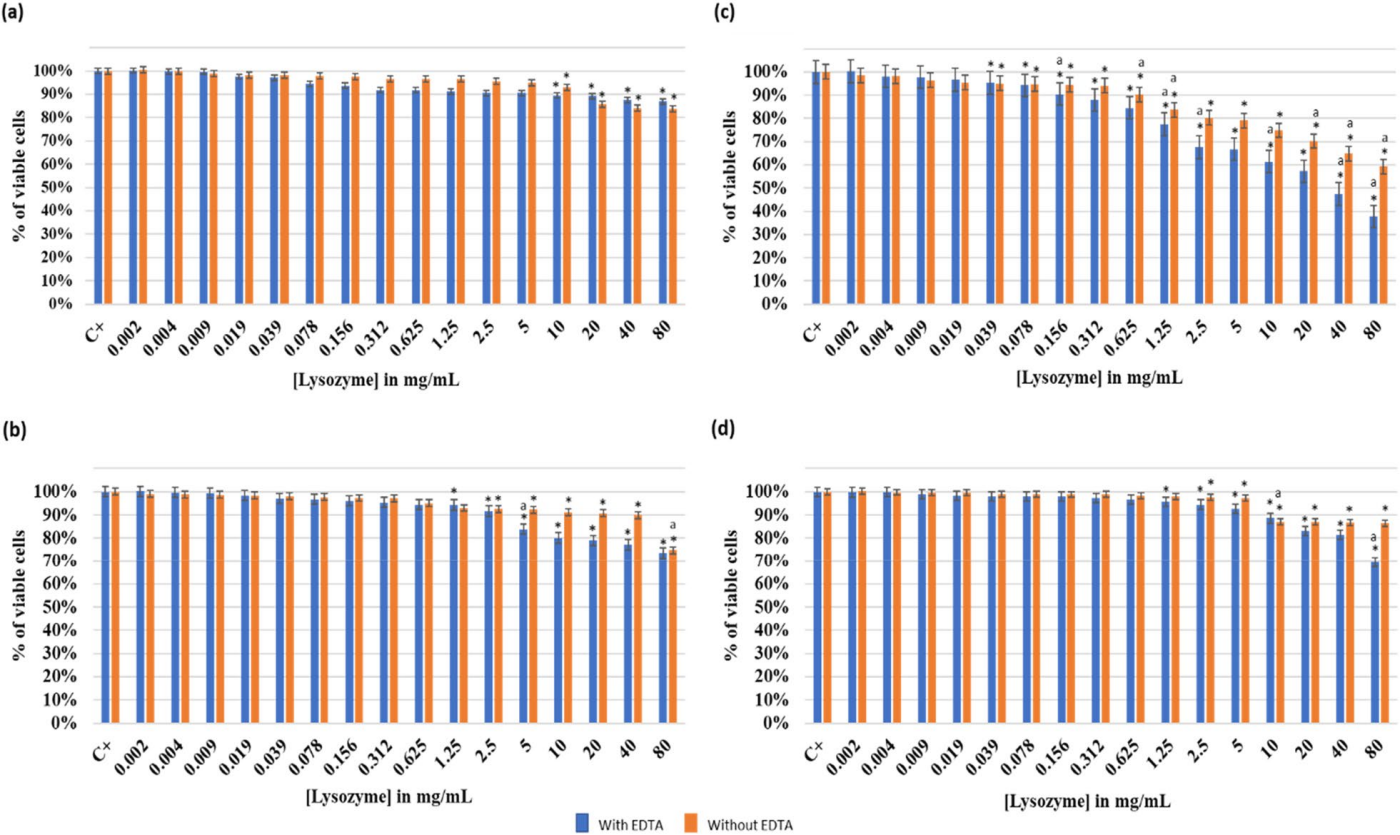
Effect of lysozyme with and without EDTA (1 mM) on the co-culture of *Lactobacillus rhamnosus* GG and *Staphylococcus epidermidis* 444 at different pH: **a** pH 2; **b** pH 6; **c** pH 7.5 and **d** pH 8.5. The results are mean values of three replicates. OD_600_ of the positive controls: C^+^(a) = 0.37; C^+^(b) = 1.15; C^+^(c) = 1.19;C^+^(d) = 1.40. (*) indicates a significant difference (P < 0.05) between each test and the positive control (without lysozyme). The lowercase letter **a** indicates a significant difference (P < 0.05) between each test and its preceding. Error bars represent the SD (standard deviation)

The antimicrobial activity of the lysozyme with and without 1 mM EDTA on co-culture at different pH is highlighted in Fig. 3.

According to Fig. 3a, at pH 2, results were in accordance with when testing each strain singly. At 80 mg/mL of lysozyme, the percentages of cells viability were 87% (with EDTA) and 84% (without EDTA). Figure 3b showed a significant decrease (P < 0.05) from 100% (positive control) to 73% (with EDTA) and 75% (without EDTA) of the viable cells at a lysozyme concentration of 80 mg/mL and a pH of 6. However, at a pH of 7.5 (Fig. 3c), an optimal antimicrobial effect was detected. Thus, at 80 mg/mL of lysozyme, a gradual decrease of viable cells occurred till reaching respectively 38% (with EDTA) and 59% (without EDTA). In contrast to the other tested pH levels, a MIC was observed at 80 mg/mL and the IC_50_ was at 26 mg/mL of lysozyme (with EDTA). The obtained results were quite similar to those of lysozyme performed with *L. rhamnosus* GG. At a pH of 8.5 (Fig. 3d), the effect of the lysozyme has become weak, thus a slower decrease (P < 0.05) in viable cells was observed and a reduction to 70% (with EDTA) and 86% (without EDTA) was observed at a lysozyme concentration of 80 mg/mL.

Despite the significant results obtained by increasing lysozyme concentration on the inhibition of the tested strains, the absence of minimum bactericidal concentrations (MBC) was remarkable at all pH levels.

#### Effect of oxytetracycline hydrochloride with and without lysozyme-EDTA mixture on planktonic *Lactobacillus rhamnosus* GG

**Fig. 4:**
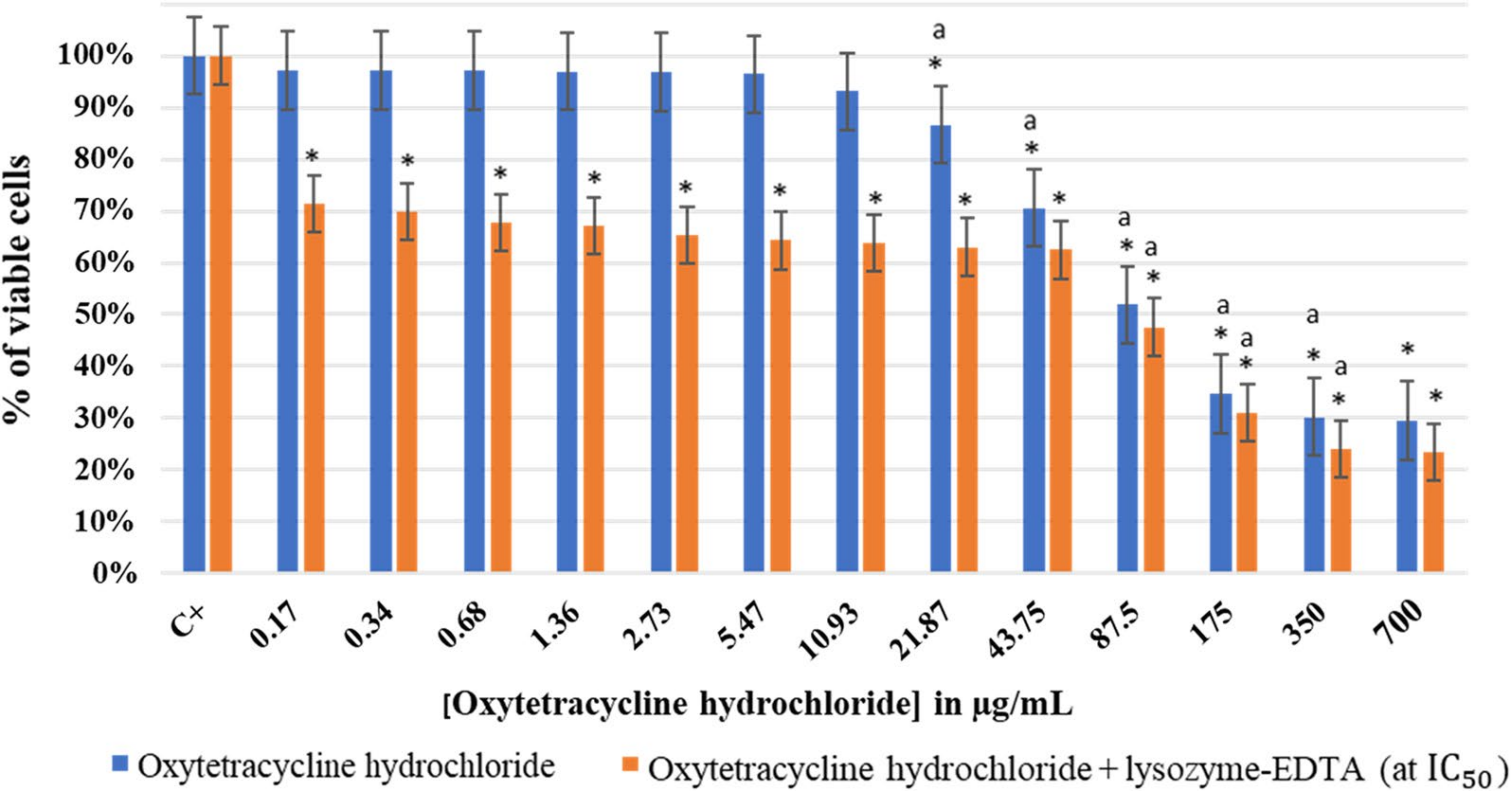
Effect of oxytetracycline hydrochloride with and without lysozyme-EDTA (at IC_50_) on planktonic *Lactobacillus rhamnosus* GG at pH 7.5. OD_600_ of negative control (oxytetracycline hydrochloride) = 0.32, OD_600_ of 700 μg/mL of oxytetracycline hydrochloride with lysozyme-EDTA (at IC_50_) = 0.38. The results are mean values of three replicates. (*) indicates a significant difference (P < 0.05) between each test and the positive control (oxytetracycline hydrochloride). The lowercase letter **a** indicates a significant difference (P < 0.05) between each test and its preceding. Error bars represent the SD (standard deviation)

The effect of using singly oxytetracycline hydrochloride or mixing it with lysozyme-EDTA (at IC_50_) on *L. rhamnosus* GG at pH 7.5 is shown in Fig. 4.

The oxytetracycline hydrochloride significant effect (P < 0.05) started to be observed at a concentration of 21.87 µg/mL. The antibiotic effect kept increasing while its concentration increased, where 69% of viable *L. rhamnosus* GG were inhibited at a concentration of 700 µg/mL and a MIC was observed. Nevertheless, after adding 30 mg/mL of lysozyme with EDTA (at IC_50_), the percentage of cell viability started to drop down from lower antibiotic concentrations (0.17 µg/mL). Hence, at this concentration a significant reduction (P < 0.05) in the percentage of viable cells (up to 71%) was observed. This reduction continued gradually till reaching 23% at an antibiotic concentration of 700 μg/mL after the addition of lysozyme and EDTA. The IC_50_ was reduced from 152 μg/mL when singly using oxytetracycline hydrochloride to 90 μg/mL after lysozyme addition.

After performing the MBC test, it was noted that in the absence of the lysozyme, oxytetracycline hydrochloride could not inhibit *L. rhamnosus* GG at 700 μg/mL. When plating the same concentration of antibiotic with lysozyme-EDTA (at IC_50_) no colonies of *L. rhamnosus* GG were observed and an MBC was detected (Additional file 1: Fig. S3). The OD_600_ at 700 μg/mL of oxytetracycline hydrochloride with lysozyme-EDTA (at IC_50_) was of 0.38, which was similar to that of the negative control (OD_600_ oxytetracycline hydrochloride).

#### Effect of oxytetracycline hydrochloride with and without lysozyme-EDTA mixture on planktonic *Staphylococcus epidermidis* 444

**Fig. 5:**
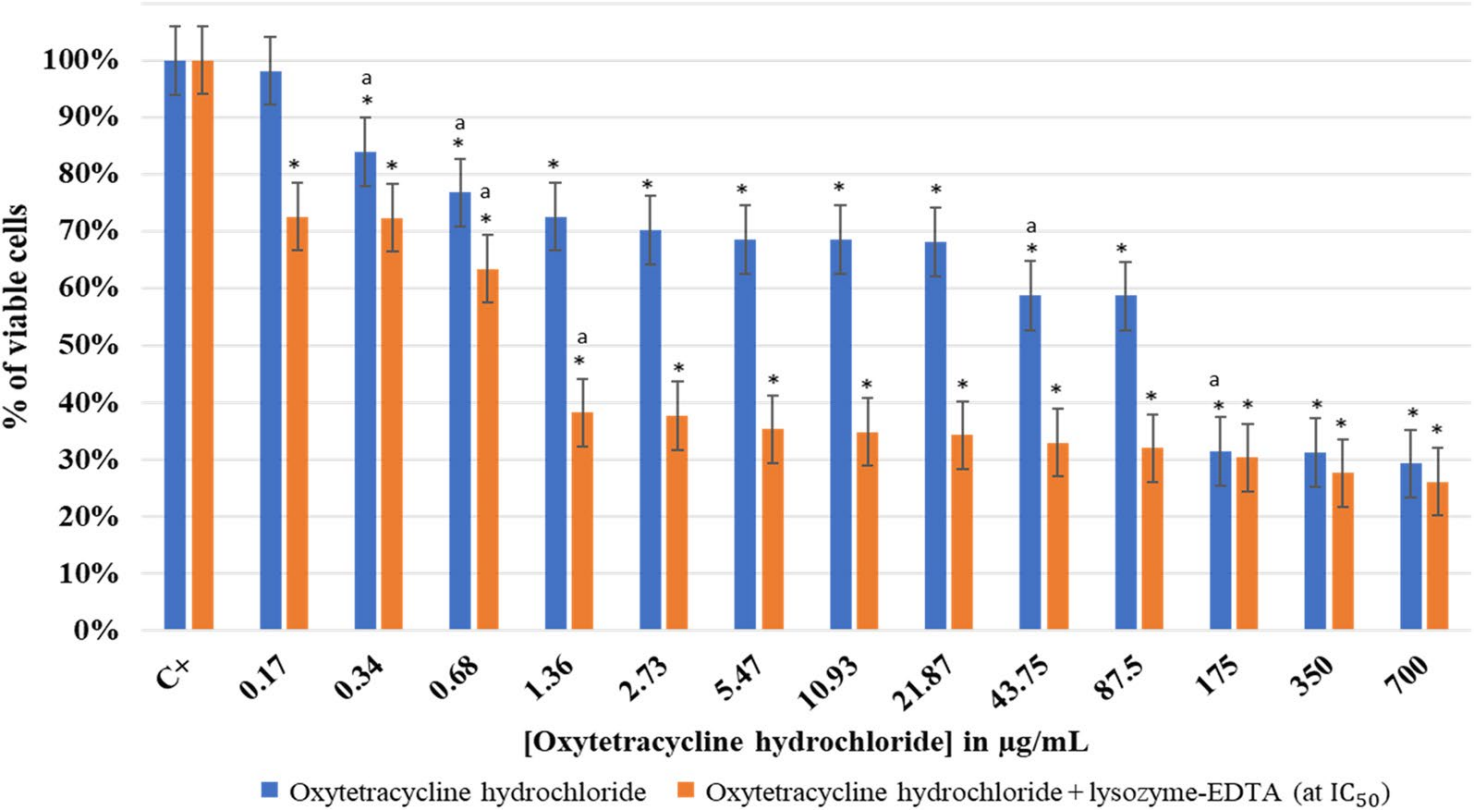
Effect of oxytetracycline hydrochloride with and without lysozyme-EDTA (at IC_50_) on planktonic *Staphylococcus epidermidis* 444 at pH 7.5. OD_600_ negative control (oxytetracycline hydrochloride) = 0.32, OD_600_ of 700 μg/mL of oxytetracycline hydrochloride with lysozyme-EDTA (at IC_50_) = 0.4. The results are mean values of three replicates. (*) indicates a significant difference (P < 0.05) between each test and the positive control (oxytetracycline hydrochloride). The lowercase letter (a) indicates a significant difference (P < 0.05) between each test and its preceding. Error bars represent the SD (standard deviation)

The effect of using singly oxytetracycline hydrochloride or mixing it with lysozyme-EDTA (at IC_50_) on *S. epidermidis* 444 at pH 7.5 is shown in Fig. 5.

As seen in Fig. 5, at a concentration of 700 µg/mL, oxytetracycline hydrochloride significantly reduced the bacterial growth of viable *S. epidermidis* 444 (P < 0.05) up to 29% without being able to totally inhibit their growth. A MIC was visually observed at this concentration (700 µg/mL). One the other hand, the addition of 18 mg/mL of lysozyme-EDTA (at IC_50_) to this antibiotic revealed a significant drop (P < 0.05) in the percentage of viability from 100 to 26% when using 700 µg/mL of antibiotic (with lysozyme). The IC_50_ was observed at 130 μg/mL (without lysozyme) and at 1.22 μg/mL (with lysozyme) of oxytetracycline hydrochloride. Therefore, we hypothesize that the lysozyme has made *S. epidermidis* 444 more vulnerable to oxytetracycline hydrochloride, even with its broad-spectrum antibiotic profile. Consequently, lysozyme-EDTA mixture increased antibiotic killing of *S. epidermidis* 444 even after showing a certain resistance to high antibiotic concentrations (Additional file 1: Fig. S4). Similarly, an MBC was observed when lysozyme-EDTA mixture (at IC_50_) was added to 700 µg/mL of oxytetracycline hydrochloride.

#### Effect of oxytetracycline hydrochloride with and without lysozyme-EDTA mixture on a co-culture of planktonic *Lactobacillus rhamnosus* GG and *Staphylococcus epidermidis* 444

**Fig. 6:**
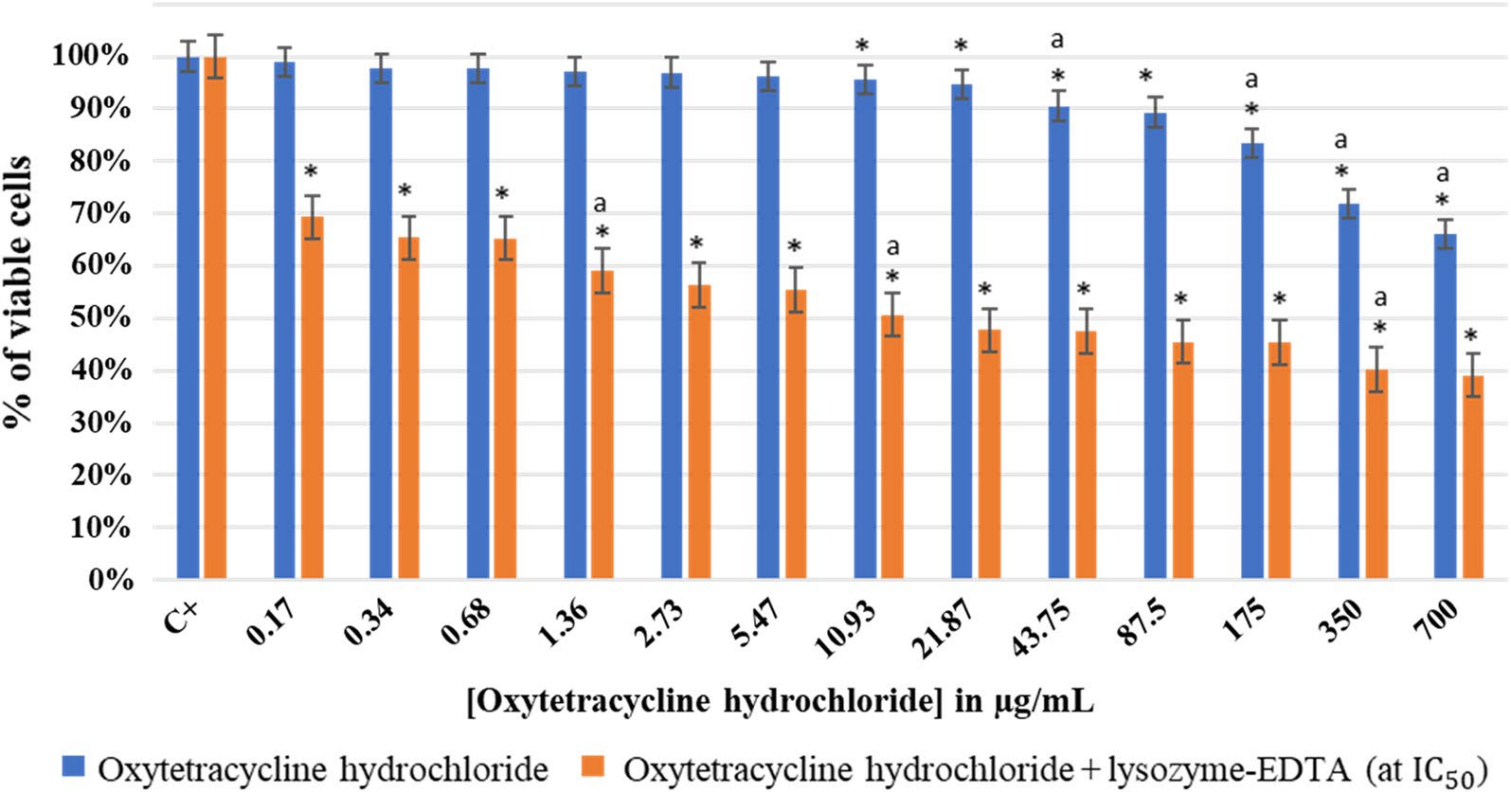
Effect of oxytetracycline hydrochloride with and without lysozyme-EDTA (at IC_50_) on the planktonic co-culture of *Lactobacillus rhamnosus* GG and *Staphylococcus epidermidis* 444. OD_600_ negative control (oxytetracycline hydrochloride) = 0.32. OD_600_ of 700 μg/mL of oxytetracycline hydrochloride with lysozyme-EDTA (at IC_50_) = 0.30. The results are mean values of three replicates. (*) indicates a significant difference (P < 0.05) between each test and the positive control (oxytetracycline hydrochloride). The lowercase letter **a** indicates a significant difference (P < 0.05) between each test and its preceding. Error bars represent the SD (standard deviation)

Figure 6 shows the effect of oxytetracycline hydrochloride on a co-culture of *L. rhamnosus* GG and *S. epidermidis* 444 planktonic strains after 24 h of incubation. The percentage of cells viability decreased progressively to 66% at an antibiotic concentration of 700 μg/mL at which a MIC was observed. A higher resistance to antibiotic was observed when the two strains have been co-cultured compared to when singly tested. When 26 mg/mL of lysozyme-EDTA (at IC_50_) were added to oxytetracycline hydrochloride, a significant and gradual reduction of the viable cells (P < 0.05) toward reaching 39% was observed. Thus, an IC_50_ was detected at an antibiotic concentration of 10.93 μg/mL. The positive effect of lysozyme combined to EDTA on the co-cultured strains is well noticeable. Interestingly, the minimum bactericidal concentrations (MBC) were absent at 700 µg/mL of oxytetracycline hydrochloride when lysozyme and EDTA were not added (Additional file 1: Fig. S5).

### Antimicrobial assays for bacterial biofilms

The minimal concentration to eradicate the formed biofilm by 50% (MCBEC_50_) was assayed where the optical density (OD_570_) was measured after 24 h of incubation. The obtained OD_570_ was converted into a percentage of biofilm formation.

#### Effect of increasing lysozyme concentration on *Lactobacillus rhamnosus *GG*, Staphylococcus epidermidis *444*,* and co-culture biofilm

**Fig. 7:**
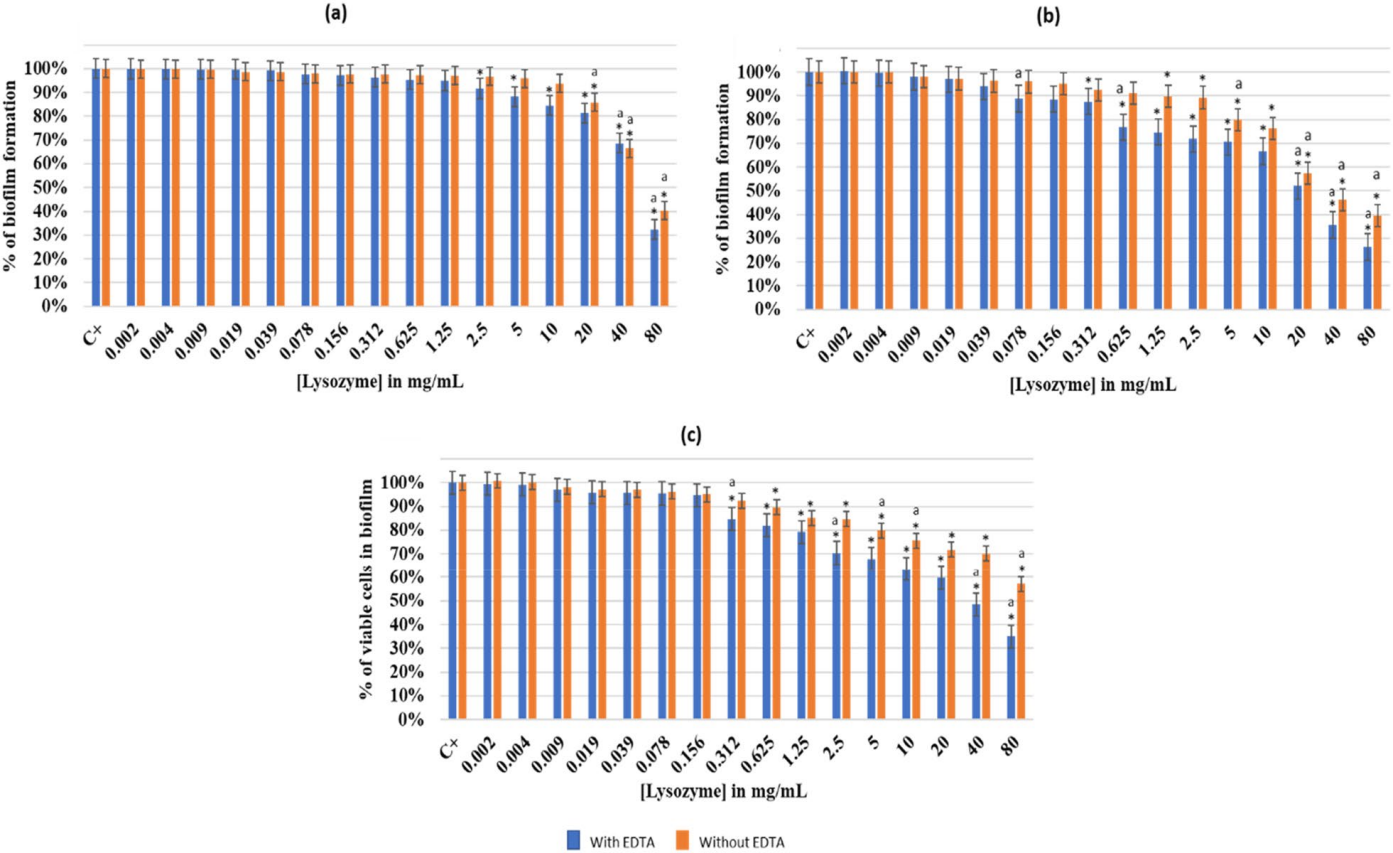
Effect of Lysozyme with and without EDTA (1 mM) on different biofilms at pH7.5. **a** *Lactobacillus rhamnosus* GG, **b** *Staphylococcus epidermidis* 444, and **c** co-culture of both strains. The results are mean values of three replicates. (*) indicates a significant difference (P < 0.05) between each test and the positive control (without lysozyme). The lowercase letter **a** indicates a significant difference (P < 0.05) between each test and its preceding. Error bars represent the SD (standard deviation)

Figure 7 shows the effect of lysozyme (with and without EDTA) on different biofilms at pH 7.5.

When adding lysozyme with EDTA to *L. rhamnosus* GG biofilm (Fig. 7a), a gradual increase in the biofilm eradication was observed. The biofilm formation significantly decreased (P < 0.05) and reached 32% (with EDTA) and 40% (without EDTA) at a lysozyme concentration of 80 mg/mL. On the other hand, a gradual decreased by around 50% in the formed biofilm of *S. epidermidis* 444 was observed when using 20 mg/mL of lysozyme (with EDTA). Similarly, as shown in Fig. 7b, an eradication of 74% of the same biofilm was observed at 80 mg/mL of lysozyme (with EDTA). In the absence of EDTA, a less significant eradication (P < 0.05) of the formed biofilm was observed. Thus, at a concentration of 80 mg/mL of lysozyme, the biofilm formation by *S. epidermidis* 444 was of 39%. The co-culture biofilm of both strains shown in Fig. 7c, revealed a gradual decrease when increasing the lysozyme concentration. This gradual decrease in biofilm formation reached up to 35% (with EDTA) and 57% (without EDTA) at 80 mg/mL of lysozyme. The minimal complete biofilm eradication concentrations MCBEC_50_ were obtained by following a series of serial dilutions. 50, 26, and 30 mg/mL of lysozyme (with EDTA) were respectively MCBEC_50_ of *L. rhamnosus* GG, *S. epidermidis* 444*,* and the co-culture of both strains. The obtained lysozyme concentrations (with EDTA) will be added to different antibiotic concentrations to evaluate their effect on increasing biofilm eradication.

#### Effect of oxytetracycline hydrochloride with and without lysozyme-EDTA mixture on *Lactobacillus rhamnosus *GG*, Staphylococcus epidermidis *444*,* and co-culture biofilm

**Fig. 8:**
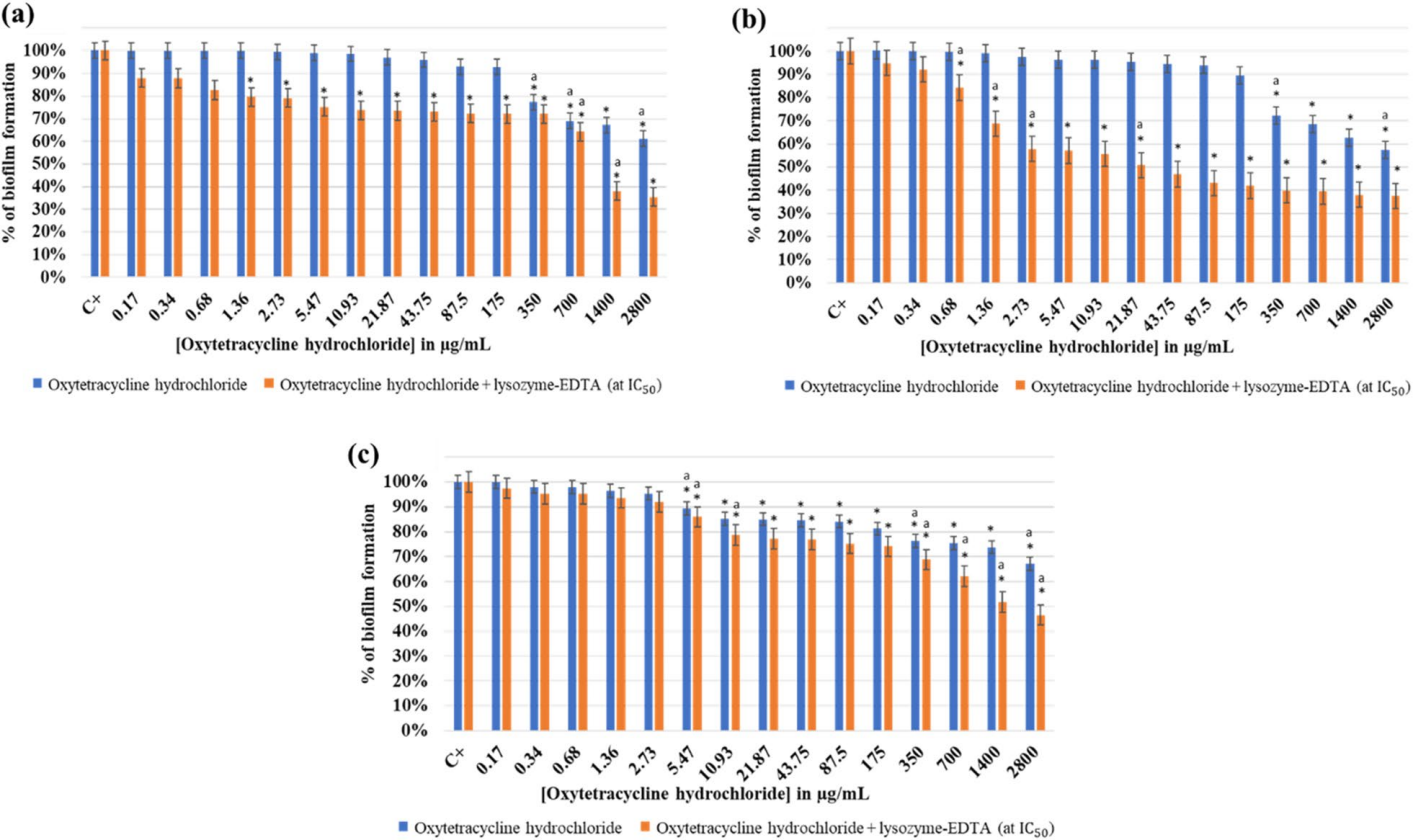
Effect of oxytetracycline hydrochloride with and without lysozyme-EDTA (at MCBEC_50_) on different biofilms at pH7.5. **a** *Lactobacillus rhamnosus* GG, **b** *Staphylococcus epidermidis* 444, and **c** co-culture of both strains. The results are mean values of three replicates. (*) indicates a significant difference (P < 0.05) between each test and the positive control (oxytetracycline hydrochloride). The lowercase letter **a** indicates a significant difference (P < 0.05) between each test and its preceding. Error bars represent the SD (standard deviation)

The effect of oxytetracycline hydrochloride on *L. rhamnosus* GG biofilm eradication with and without the addition of lysozyme and EDTA at pH 7.5 is shown in Fig. 8a.

The biofilm of *L. rhamnosus* GG showed a high resistance against the used antibiotic. Thus, the percentage of biofilm formation was constant until a significant decrease (P < 0.05) started at an antibiotic concentration of 350 μg/mL. Notably, when 50 mg/mL of lysozyme with EDTA (at MCBEC_50_) had been added to the antibiotic, the biofilm was eradicated by 12% starting from the lowest concentration (0.17 μg/mL). This eradication continued gradually until reaching 38% and 35% in the presence of 1400 μg/mL and 2800 μg/mL of oxytetracycline hydrochloride. A MCBEC_50_ was observed after adding 1144 μg/mL of oxytetracycline hydrochloride to lysozyme-EDTA mixture. However, lysozyme with EDTA induced higher eradication when added to the antibiotic.

The effect of oxytetracycline hydrochloride on *S. epidermidis* 444 biofilm eradication with and without the addition of lysozyme and EDTA at pH 7.5 is shown in Fig. 8b.

Hence, oxytetracycline hydrochloride weakly affected the biofilm eradication of *S. epidermidis* 444 up to a concentration of 350 μg/mL, where a significant (P < 0.05) drop of 28% was observed. The increase in the biofilm eradication continued gradually to reach its optimum of 43% at 2800 μg/mL oxytetracycline hydrochloride. On the other hand, when the antibiotic was coupled with 26 mg/mL of lysozyme with EDTA (at MCBEC_50_), a much greater eradication was observed even with lower antibiotic concentrations. Thus, at an antibiotic concentration of 2.73 μg/mL, the percentage of biofilm eradication was of 2% compared to 48% after the addition of lysozyme-EDTA mixture. Hence, a 21-fold of eradication increase was noticed. Significantly, the decrease in biofilm formation when increasing the antibiotic concentration (P < 0.05) caused a reduction of the formed *S. epidermidis* biofilm by 63% at an antibiotic concentration of 2800 μg/mL with the addition of 26 mg/mL of lysozyme mixed with EDTA. Accordingly, a new MCBEC_50_ was observed at 24 μg/mL of oxytetracycline hydrochloride after adding lysozyme and EDTA.

The effect of oxytetracycline hydrochloride on co-culture of *L. rhamnosus* GG and *S. epidermidis* 444 biofilm eradication with and without the addition of lysozyme and EDTA at pH 7.5 is shown in Fig. 8c.

The biofilm formed of the two strains showed high resistance at low concentrations of oxytetracycline hydrochloride. Thus, oxytetracycline hydrochloride weakly affected the co-culture biofilm eradication up to a concentration of 350 μg/mL, where a significant (P < 0.05) drop of 22% was observed. This eradication increased gradually to finally reach 33% (P < 0.05) at an antibiotic concentration of 2800 μg/mL. However, when the antibiotic was combined to 30 mg/mL of lysozyme with EDTA (at MCBEC_50_), a substantially higher level of eradication was observed starting at an antibiotic concentration of 10.93 μg/mL. The biofilm was eradicated by 22% starting with an antibiotic concentration of 10.93 μg/mL. This eradication continued to gradually increase until reaching 49% and 54% in the presence of 1400 μg/mL and 2800 μg/mL of oxytetracycline hydrochloride respectively. Accordingly, a new MCBEC_50_ was observed at a concentration of 1464 μg/mL of oxytetracycline hydrochloride after the addition of lysozyme and EDTA.

### Comparison of IC_50_, MIC, MBC and MCBEC_50_ values

The IC50, MIC, MBC and MCBEC50 values obtained from the different tests performed are shown in Table 1. From the above results it may be noticed that *S. epidermidis* 444 was more sensitive to lysozyme than *L. rhamnosus* GG*.* Indeed, the addition of EDTA to the lysozyme induced a significant improvement in its effect. In addition, the activity of the lysozyme varied widely between the different tested pH levels. However, the lysozyme activity was almost absent at a pH of 2 and quite variable between 6 and 8.5. Indeed, lysozyme’s activity seems to be more effective at a pH of 7.5. Similarly, when lysozyme-EDTA mixture was combined to oxytetracycline hydrochloride a better biofilm eradication of the tested strains was observed.

### Comparison of FIC_50_ values

Table 2 shows the effect of combining lysozyme-EDTA to oxytetracycline hydrochloride on the tested bacterial strains.

**Table 2:** FIC_50_ and ƩFIC_50_ for combinations of lysozyme-EDTA and oxytetracycline hydrochloride at pH 7.5 against planktonic *Lactobacillus rhamnosus* GG, *Staphylococcus epidermidis* 444 and co-culture strains

Strains	FIC_50_ (lysozyme-EDTA)	FIC_50_ (Oxytetracycline hydrochloride)	FIC_50_ index (ƩFIC_50_)	Effect
*L. rhamnosus* GG	3	0.59	3.59	Indifference
*S. epidermidis* 444	0.06	0.009	0.069	Synergism
Co-culture strains	0.4	< 0.015	< 0.415	Synergism

For *L. rhamnosus* GG, an indifferent effect when combining lysozyme-EDTA and oxytetracycline hydrochloride was observed where ΣFIC_50_ (= 3.59) was between 1 and 4. Thus, this indifference is mainly due to lysozyme-EDTA mixture that didn’t affect much *L. rhamnosus* GG (FIC_50_ = 3) as compared to oxytetracycline (FIC_50_ = 0.59). However, when combining antibiotic to lysozyme-EDTA mixture a high synergic effect (ΣFIC_50_ ≤ 0.5) was observed on *S. epidermidis* 444 indicating a remarkable increase in bacterial inhibition. Furthermore, same synergic results but with a lower rate were observed when the two strains where co-cultured.

## Discussion

The obtained results were in accordance with other studies revealing that *L. rhamnosus* GG and *S. epidermidis* 444 can survive in a harsh environment. Thus, they can resist pH values as low as 2.5 but with a low survival rate and poor proliferative capacity (Nishiyama et al. 2016; Ferraboschi and Ciceri 2021). Therefore, this may justify the decrease in the survival rate in the stomach. It is important to mention that the lysozyme maintains its activity in a wide pH range going from an acidic pH 6 to a basic pH 9, which may explain its minimal effect at pH 2 and its optimum at pH 7.5, then its decrease at pH 8.5 (Wang et al. 2020). Also, it is known that the proliferation of *L. rhamnosus* GG becomes increasingly important throughout the small intestine (pH 7.5) (Kosecka-Strojek et al. 2020), and this was revealed by the increase in the OD_600_ as mentioned before. In addition, this pH was the most favorable for the formation of *L. rhamnosus* GG biofilm, indicating a high cell adhesion ability, especially in the small intestine (von Rosenvinge et al. 2013). Furthermore, it can be concluded that EDTA enhanced the effect of lysozyme when approaching higher lysozyme concentrations. The study conducted by Boland et al. in 2004 was in accordance with the obtained results, where EDTA increased the lysozyme inhibitory action against *E. coli* strains (Boland et al. 2004). Accordingly, the disruption of the lipopolysaccharide structure of the bacteria's outer membrane is mainly due to chelating divalent cations (Mg^2+^, Ca^2+^) from their binding sites and making it more permeable to other substances (Reidmiller et al. 2006), in our case lysozyme. It was also previously reported that *L. rhamnosus* GG may have an antimicrobial effect against different pathogenic bacteria (Zhang et al. 2018). In the conducted study, the effect of *L. rhamnosus* GG on *S. epidermidis* 444 was omitted since the control that is used for comparison purpose is also a co-culture of both strains. Thus, what is observed is only the effect of the antimicrobial agents.

Oxytetracycline hydrochloride is known as a bacteriostatic antibiotic, with a broad-spectrum, that inhibit the protein synthesis (Grenni et al. 2017), thus this may explain the lack of a complete killing effect even at high concentrations. In fact, the modes of action of antimicrobials are quite limited, either by inhibiting the cell wall, DNA, proteins or folic acid synthesis or by disrupting the osmotic integrity (Reygaert 2018). *L. rhamnosus* GG and *S. epidermidis* 444 may developed a certain resistance to antibiotics in the gut, which is known to be a crowded bacterial environment. When adding lysozyme and EDTA to oxytetracycline hydrochloride, an MBC was achieved as shown previously. Thus, bacterial cell wall lyses may be behind this increased inhibition that is possibly due to a better antibiotic access to the targeted cells(Ferraboschi and Ciceri 2021). Therefore, a synergistic effect took place where the antibiotic was able to better attack the proteins after a cell wall disruption by lysozyme. Accordingly, lysozyme provided to oxytetracycline hydrochloride a bactericidal effect in addition to its known bacteriostatic effect.

Furthermore, EDTA played an important role in the eradication of the biofilm by amplifying considerably the effect of lysozyme. The observed results were in accordance with another study conducted by Liu et al. in 2018 that evaluated the effectiveness of EDTA on biofilm eradication, where EDTA helped in chelating Mg^2+^ and Ca^2+^ of the biofilm's EPS. Thus, EDTA made the matrix more permeable to other agents (Fangning Liu 2018). As previously observed in planktonic strains, lysozyme induced biofilm eradication and EDTA enhanced this eradication. Moreover, *L. rhamnosus* GG was more resistant to lysozyme than *S. epidermidis* 444. The biofilm of the co-culture strains had a slightly more crucial resistance against the lysozyme, which may be due to the resistance of *L. rhamnosus* GG against this enzyme. These results were consistent with the planktonic’s results, since it could be referred to the effect of lysozyme that facilitates the path of the antibiotic and increase the eradication of the formed biofilm.

The bacterial wall of the bacillary forms, in our case *L. rhamnosus* GG is more compact since the interpeptide bonds are directly linked (Ghuysen 1960). In contrast, the wall is more loose in spherical forms, such as *S. epidermidis* 444 where the interpeptide bonds are long (Ghuysen 1960). Thus, this may explain why lysozyme can more easily hydrolyze the wall of *S. epidermidis* 444 in comparison to *L. rhamnosus* GG. Moreover, several previous studies have demonstrated the ability of lysozyme to prevent biofilm formation (Leitch and Willcox 1999; Ferraboschi and Ciceri 2021). Hence, a concentration of 30 μg/mL was able to decrease the biofilm-forming capacities of a wide spectrum of hospital and reference strains of microorganisms such as *Gardnerella vaginalis*, *Staphylococcus aureus*, *Staphylococcus epidermidis* and *Pseudomonas aeruginosa* (Hukić et al. 2018). Therefore, we can hypothesize that low lysozyme concentrations may be recommended for continuous daily intake as a preventive agent against the formation of pathogenic biofilms in the gut. Thus, immunocompromised patients and other vulnerable categories may benefit from its beneficial effect. To note that *S. epidermidis* 444 tends to form mature biofilms to render their strains more resistant to antibiotics and host defense systems (Akinkunmi et al. 2014; Tamburini et al. 2018). However, in case of a need for an inhibition, killing of or eradication of S. *epidermidis* biofilms, a higher concentration of lysozyme mixed with EDTA should be added to the used antibiotic. In addition, using a mixture of lysozyme and EDTA may not be harmful to the gut microbiota. The observed synergism indicates the ability of this antibiotic-lysozyme-EDTA mixture to affect *S. epidermidis* 444 even when present in a co-culture environment such as the gut. For the co-culture strains, the difference relies possibly in the presence of *L. rhamnosus* GG that is not highly affected by the lysozyme-EDTA mixture.

In this study, the ability of the lysozyme to inhibit the growth of *L. rhamnosus* GG and *S. epidermidis* 444 and to eradicate their biofilms in the gut microbiota was investigated. An optimum bacterial survival and antimicrobial activity were observed at pH 7.5. Lysozyme with EDTA inhibited 50% of the bacterial growth of *L. rhamnosus* GG, *S. epidermidis* 444 and the co-culture of both strains respectively at 30 mg/mL, 18 mg/mL, and 26 mg/mL. By adding lysozyme-EDTA at IC_50_ and MCBEC_50_ concentrations to oxytetracycline hydrochloride, an increase in bacterial inhibition and biofilm eradication was observed. A synergic effect (ΣFIC_50_ ≤ 0.5) between lysozyme-EDTA mixture and oxytetracycline hydrochloride was detected against *S. epidermidis* 444. Nevertheless, the choice of oxytetracycline was made on purpose to use a broad-spectrum bacteriostatic antibiotic in which *S. epidermidis* 444 is known to be resistant to. Thus, the obtained results revealed the effectiveness of the developed mixture. At pH 7.5, a 100% inhibition of bacterial growth and a 70% of biofilm eradication were achieved. To note, this study may also be applicable to other microbiome such as vaginal microbiota, where the same both strains coexist. At the end, the development of new drugs based on mixing antibiotic to lysozyme and EDTA may be a turning point toward limiting antibiotic resistance and increasing its performance. Hence, more studies should be performed in vitro and in vivo using different pathogenic strains and antibiotics in order to further study the efficiency of this antimicrobial mixture on animal and human microbiota.

## Supplementary Information

**Additional file 1: Fig. S1**. Growth curves of *L. rhamnosus* GG in MRS medium. **Fig. S2**. Growth curves of *S. epidermidis* 444 in MHB medium. **Fig. S3**. Effect of 700 µg/mL of oxytetracycline hydrochloride on planktonic *Lactobacillus rhamnosus* GG. **Fig. S4**. Effect of 700 µg/mL of oxytetracycline hydrochloride on planktonic *Staphylococcus epidermidis* 444. **Fig. S5**. Effect of 700 µg/mL of oxytetracycline hydrochloride on a planktonic co-culture of *Lactobacillus rhamnosus* GG and *Staphylococcus epidermidis* 444 cultured on selective mediums: (A) MRS for *L. rhamnosus* GG; (B) BP for *S. epidermidis* 444.

## Data Availability

Authors can confirm that all relevant data are included in the article and/or its additional files.
