# Supplementary material for: Lactobacillus rhamnosus and Staphylococcus epidermidis in gut microbiota: in vitro antimicrobial resistance
Source: AMB Express. 2022 Oct 3;12:128. doi: 10.1186/s13568-022-01468-w (PMC9530110; doi:10.1186/s13568-022-01468-w)

**Journal name:** AMB Express

**Manuscript Title:** *Lactobacillus rhamnosus* and *Staphylococcus epidermidis* in gut microbiota: In vitro antimicrobial resistance

**The name(s) of the author(s):** Pamela Hindieh; Joseph Yaghi; André El Khoury; Ali Chokr; Ali Atoui; Nicolas Louka; Jean Claude Assaf.

**The affiliation(s) and address(es) of the author(s):**

Centre d’Analyses et de Recherche (CAR), Unité de Recherche TVA/Résistance aux Antibiotiques et Impact Industriel (RAII), Faculté des Sciences, Université Saint-Joseph de Beyrouth, Campus des sciences et technologies, Mar Roukos, Matn, Lebanon.

Laboratoire de mycologie et sécurité des aliments (LMSA), Faculté des Sciences, Université Saint-Joseph de Beyrouth, Campus des sciences et technologies, Mar Roukos, Matn, Lebanon.

**The e-mail address, telephone and fax numbers of the corresponding author:**

jeanclaude.assaf@net.usj.edu.lb

+961 70 891 797

**Fig. S1** Growth curves of *L. rhamnosus* GG in MRS medium.

**Fig. S2** Growth curves of *S. epidermidis* 444 in MHB medium.

**Fig. S3** Effect of 700 µg/mL of oxytetracycline hydrochloride on planktonic *Lactobacillus rhamnosus* GG. MBC was observed after adding 30 mg/mL of lysozyme and EDTA (1 mM) to the same concentration.

**Fig. S4** Effect of 700 µg/mL of oxytetracycline hydrochloride on planktonic Staphylococcus epidermidis 444. MBC was observed after adding 18 mg/mL of lysozyme and EDTA (1 mM) to the same concentration.

**Fig. S5** Effect of 700 µg/mL of oxytetracycline hydrochloride on a planktonic co-culture of Lactobacillus rhamnosus GG and Staphylococcus epidermidis 444 cultured on selective mediums: (A) MRS for L. rhamnosus GG; (B) BP for S. epidermidis 444. MBC was observed after adding 26 mg/mL of lysozyme and EDTA (1mM) to the same concentration.


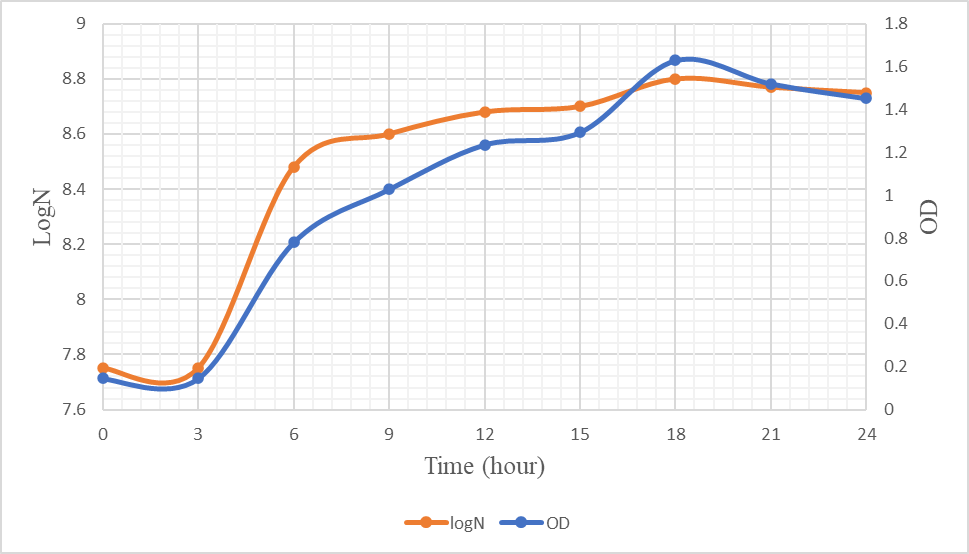


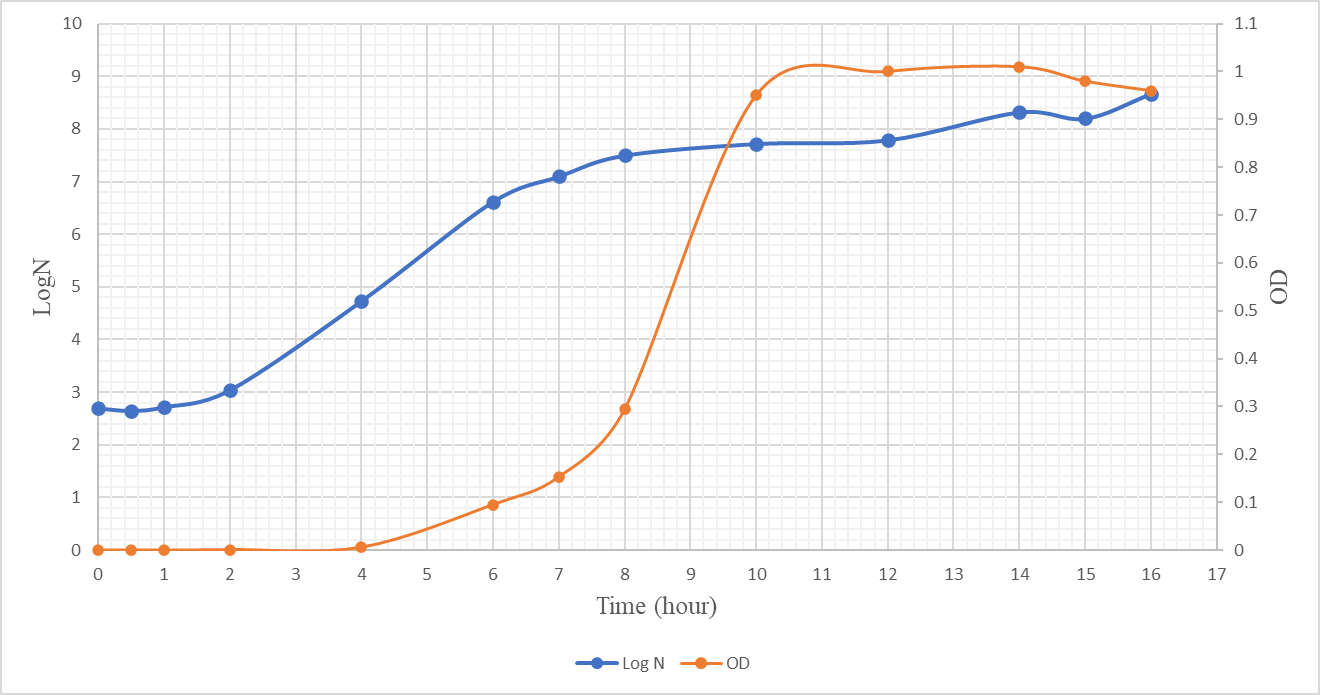


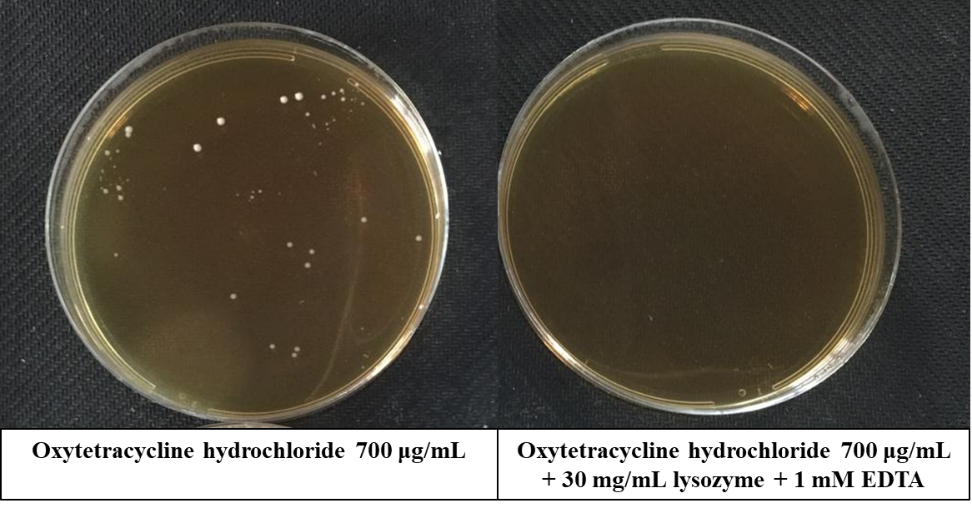


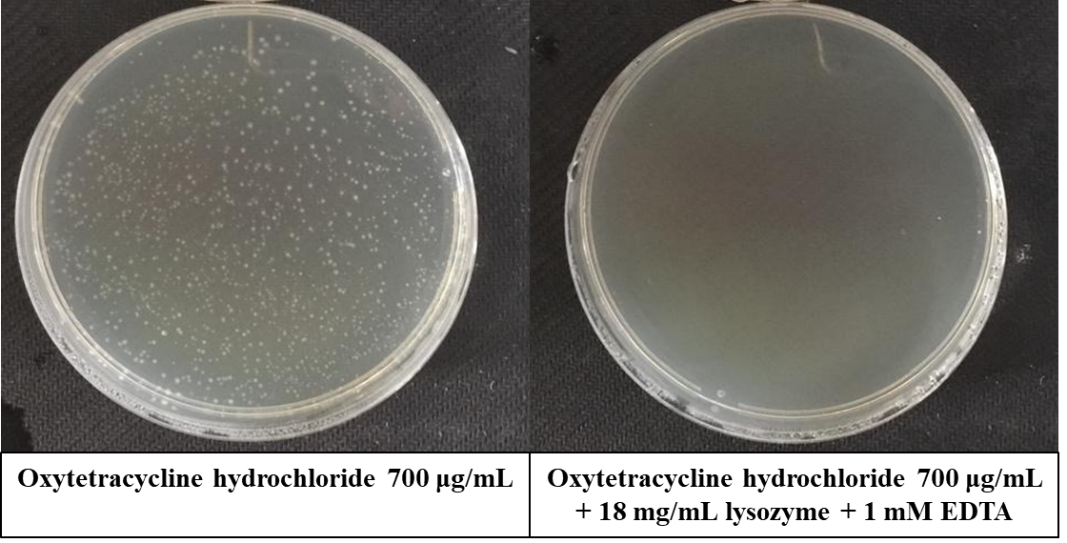


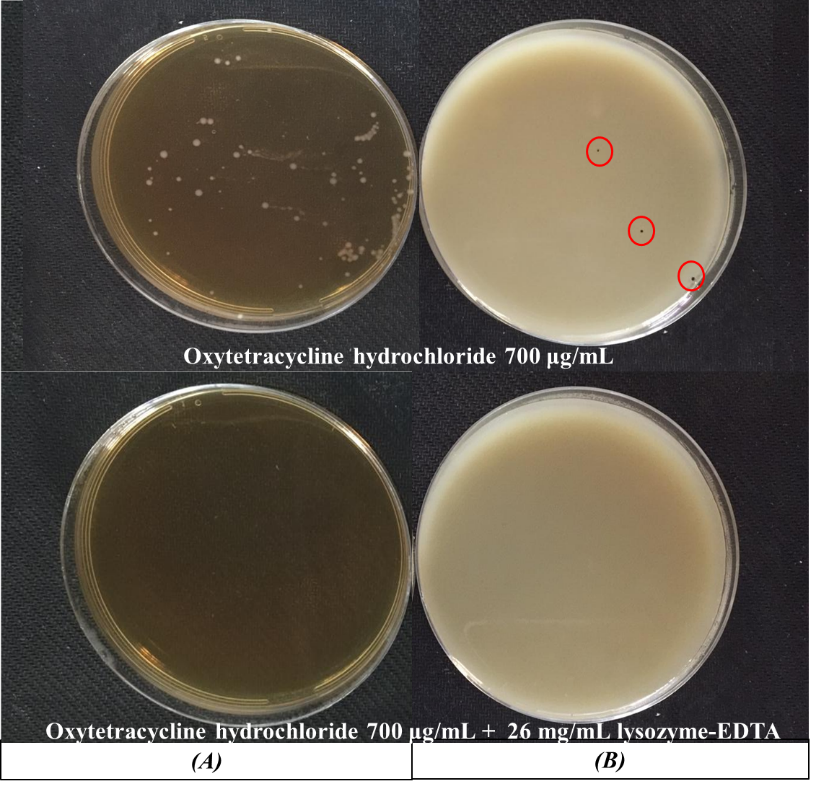

Supplement: Supplementary file 1 — Additional file 1: Fig. S1. Growth curves of L. rhamnosus GG in MRS medium. Fig. S2. Growth curves of S. epidermidis 444 in MHB medium. Fig. S3. Effect of 700 µg/mL of oxytetracycline hydrochloride on planktonic Lactobacillus rhamnosus GG. Fig. S4. Effect of 700 µg/mL of oxytetracycline hydrochloride on planktonic Staphylococcus epidermidis 444. Fig. S5. Effect of 700 µg/mL of oxytetracycline hydrochloride on a planktonic co-culture of Lactobacillus rhamnosus GG and Staphylococcus epidermidis 444 cultured on selective mediums: (A) MRS for L. rhamnosus GG; (B) BP for S. epidermidis 444. [file 13568_2022_1468_MOESM1_ESM.docx]
